# The clinical impact of drug-induced hepatotoxicity on anti-tuberculosis therapy: a case control study

**DOI:** 10.1186/s12931-019-1256-y

**Published:** 2019-12-16

**Authors:** Jin Hwa Song, Seo-Young Yoon, Tae Yun Park, Eun Young Heo, Deog Kyeom Kim, Hee Soon Chung, Jung-Kyu Lee

**Affiliations:** 1 Department of Internal Medicine, Veterans Health Service Medical Center, Seoul, Republic of Korea; 2 Division of Pulmonary and Critical Care Medicine, Department of Internal Medicine, Seoul Metropolitan Government-Seoul National University Boramae Medical Center, Seoul, Republic of Korea

**Keywords:** Tuberculosis, pulmonary, Chemical- and drug-induced liver injury, Drug-related side-effects and adverse reactions

## Abstract

**Background:**

There are limited data available on whether drug-induced hepatotoxicity (DIH) affects the clinical outcomes of tuberculosis (TB) treatment. We explored the effects of DIH on the clinical course and outcomes of pulmonary TB.

**Methods:**

In this retrospective cohort study, we included patients with culture-proven pulmonary TB treated in a tertiary hospital from 2013 to 2016. DIH was defined as proposed by the official American Thoracic Society statement. We compared the clinical outcomes of DIH and non-DIH patients.

**Results:**

Between January 1, 2013 and December 31, 2016, a total of 168 TB patients were included, and 20 (11.9%) were diagnosed with DIH. These patients were significantly older, had a higher Charlson Comorbidity Index score, exhibited more chronic liver disease, included more chronic alcoholics, and had a lower body mass index than non-DIH patients. We found no significant differences between DIH and non-DIH patients in the 2-month sputum culture conversion rate, the time to sputum culture conversion, treatment outcomes, or total treatment duration. However, the ratio of treatment interruption time to total treatment duration and the proportion of hepatotonic users were significantly higher among DIH patients.

**Conclusion:**

DIH development during TB treatment does not significantly affect the clinical outcomes of pulmonary TB. However, treatment interruption caused by DIH may increase the risks of future relapse and acquired resistance. Further study is needed.

## Background

Tuberculosis (TB) eradication remains globally challenging. TB is the most common cause of death from infectious disease; 6.30 million new TB cases and 1.7 million TB deaths were reported globally in 2016 [1]. Although long-term complex medications are required to successfully treat TB, several adverse drug events have been noted. Drug-induced hepatotoxicity (DIH) is one of the most common side-effects requiring drug interruption or modification. The incidence of DIH in patients receiving the standard drug regimen has been reported to be 2–28%, but is 10–11% in Korea [2–6]. DIH reflects direct toxicities of drug metabolites or effects thereof on immune system–mediated pathways [7, 8]. Old age, advanced TB, high-level alcohol intake, and underlying liver disease are the principal risk factors for DIH development [9, 10]. However, it is difficult to anticipate DIH because most disease is attributable to unpredictable idiosyncratic reactions. DIH may manifest as a broad spectrum of clinical features from asymptomatic elevation of liver enzyme levels to fulminant liver failure [2]; DIH may sometimes become a more serious problem than TB per se. Previous studies focused principally on the causative agents, mechanisms, and risk factors; few studies have explored the clinical outcomes of TB treatment in patients with DIH [11–14]. We explored whether DIH development during TB treatment could affect the clinical course and outcomes of TB.

## Methods

### Study design and study population

A retrospective cohort study was conducted from January 1, 2013 to December 31, 2016 at the Seoul Metropolitan Government–Seoul National University Boramae Medical Center, a tertiary referral hospital of South Korea. The cohort was assembled using the prospective registry of TB patients treated by this center. Patients with pulmonary TB satisfying the inclusion criteria were enlisted via retrospective review. The inclusion criteria were males and females aged 20 years or more, pulmonary TB as proven by acid-fast bacilli (AFB) culture of respiratory specimens, treatment with the standard regimen, and performance of 2-month follow-up sputum cultures. The standard regimen features intensive isoniazid, rifampin, ethambutol, and pyrazinamide for 2 months, followed by a maintenance phase (isoniazid and rifampin for 4 months), in line with the Korean TB guidelines [15]. Exclusion criteria were initial treatment with any regimen other than the standard regimen, relapse after previous treatment, re-treatment after failure of previous treatment, extra-pulmonary TB, drug-resistant TB, and patients whose clinical outcomes could not be ascertained because of follow-up loss, transfer to other centers, or death within 2 months. During patient selection, we excluded those exhibiting factors known to affect clinical outcomes and we sought to clearly determine the impact of DIH on the clinical course. The study was approved by the institutional review board of Seoul Metropolitan Government–Seoul National University Boramae Medical Center (No: 20170214/16–2017-21/031).

### Definition of DIH

DIH was defined according to the American Thoracic Society guideline, as follows: 1) serum aspartate aminotransferase (AST) and/or alanine aminotransferase (ALT) levels higher than 5 times the upper limit of normal (ULN; 40 IU/L); or 2) AST and/or ALT levels higher than 3 times the ULN, combined with symptoms such as easy fatigability, nausea, vomiting, abdominal pain, and/or poor oral intake; and 3) at least a 50% improvement in the liver enzyme elevations after discontinuation of anti-TB drugs [8]. If patients exhibited other causes of liver injury (re-activation or infection with hepatitis A, hepatitis B, hepatitis C, or hepatitis E virus; or human immunodeficiency virus) or excessive alcohol consumption, or took hepatotoxic medications, they were not considered to have DIH. DIH severity was graded using the 5-point scale of the Drug-Induced Liver Injury Network (DILIN) [16].

### Outcomes

The primary outcome was the proportion of patients exhibiting sputum-negative culture conversion within 2 months of treatment. The secondary outcomes were treatment duration, treatment interruption, sputum culture conversion status, time to sputum culture conversion, and treatment outcome. We obtained demographic, laboratory, microbiological, and clinical data from our electronic medical records system. Although the absolute treatment period is important when assessing the course of TB treatment, treatment duration can be extended beyond the usual time when certain factors are found at diagnosis, such as lung TB combined with TB of bone or the central nervous system, or a pulmonary cavity. Given the differences among the initial clinical situations, a comparison of absolute treatment durations may not adequately evaluate whether DIH development truly extended treatment. Thus, we examined the treatment plans prepared by clinicians when treatment commenced for all patients and calculated the ratio of the actual treatment period to the planned treatment duration. Sputum culture conversion was evaluated for both liquid and solid cultures. The outcomes of TB treatment were classified as follows: success (treatment completion or cure), failure, or death [17]. Treatment interruption was defined as the discontinuation of anti-tuberculosis therapy for any period due to drug-induced hepatotoxicity [18]. We reviewed patients who had been followed- up for more than 1 year after treatment in terms of TB recurrence.

### Statistical analysis

Normally distributed variables are shown as means ± standard deviations (SDs). The chi-squared and Fisher’s exact tests were used to compare categorical variables and the Student’s t-test used to compare continuous variables. We used multivariable logistic regression to analyze DIH risk factors. In the analysis to identify the risk factors for DIH, we used epidemiologic variables (age, sex) and underlying comorbidities related to current or future liver disease as adjustment variables. In the analysis to identify factors contributing to treatment success and sputum culture conversion, we adjusted with factors that reflect the disease extent at the time of TB diagnosis (cavitation in initial chest X-ray and smear-positivity of initial sputum specimen), in addition to the factors mentioned earlier. Odds ratios (ORs) and adjusted odds ratio (aORs) are presented with 95% confidence intervals (CIs). The time to sputum culture conversion by DIH status was estimated using the Kaplan–Meier method, and factors contributing to treatment success and sputum liquid culture conversion within 2 months were evaluated with the aid of Cox’s proportional hazards regression analysis. A *P*-value < 0.05 was considered statistically significant. All statistical analyses were performed with the aid of Stata ver. 13.0 software (StataCorp 2013, Stata Statistical Software: Release 13, College Station, TX: StataCorp LP).

## Results

### Baseline characteristics of the study population

**Fig. 1:**
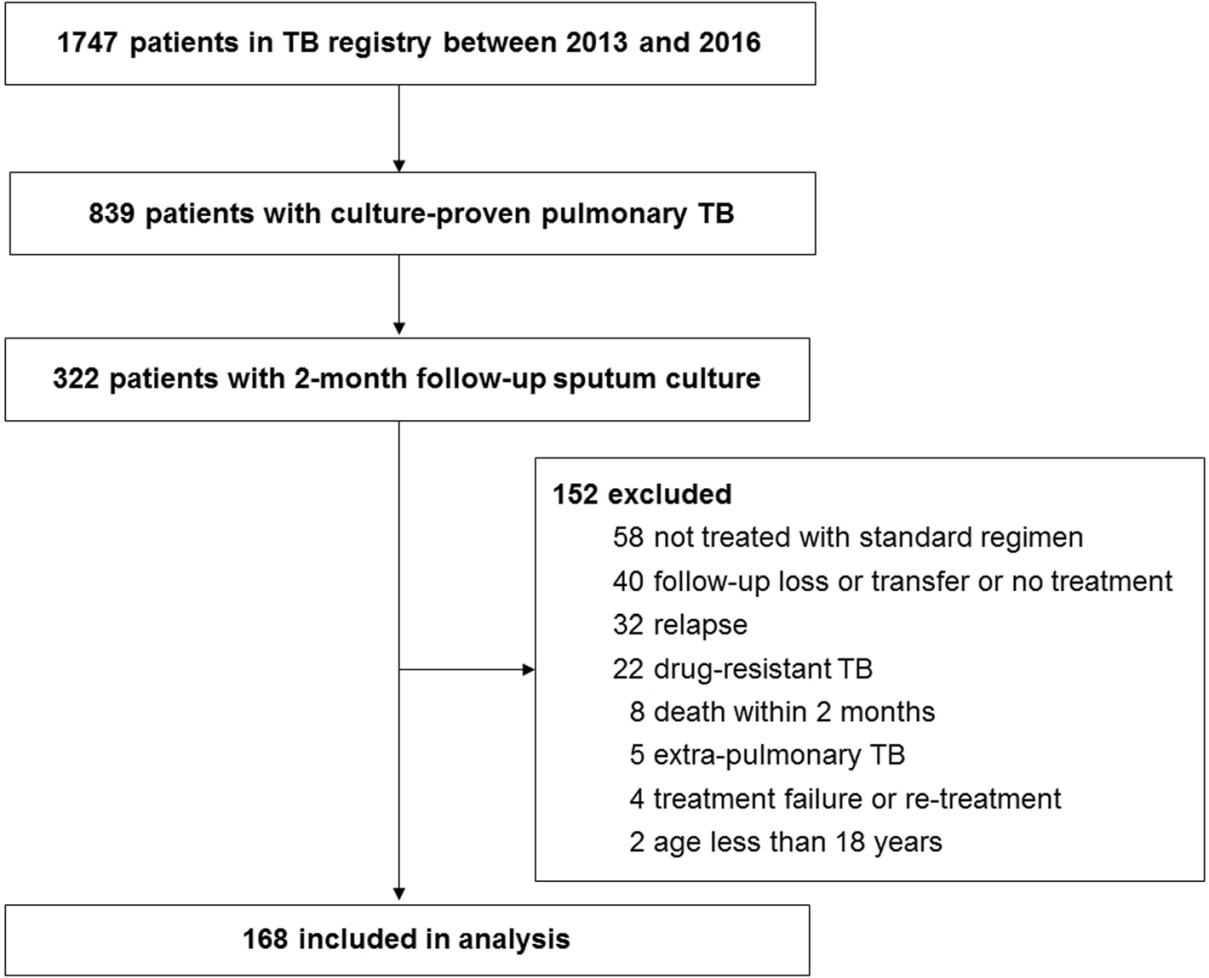
Flow diagram of the study population

Between January 1, 2013 and December 31, 2016, 1747 patients were enrolled in the TB cohort of the Seoul Metropolitan Government–Seoul National University Boramae Medical Center (Fig. 1). Of these, 839 had culture-proven pulmonary TB, and 322 underwent 2-month follow-up sputum culture. A total of 168 patients with drug-susceptible TB treated via the standard regimen were finally included in analyses. Table 1 lists their baseline demographic and clinical characteristics. The mean age was 53.4 years, and 111 patients were male (66.1%). Twenty patients developed DIH (11.9%); these patients were older than non-DIH patients (62.9 ± 17.1 vs. 52.1 ± 19.2 years; *P* = 0.018), had a significantly higher Charlson Comorbidity Index score (0.80 ± 0.83 vs. 0.43 ± 0.63; *P* = 0.042), had a more extensive history of chronic liver disease (25 vs. 6.1%, *P* = 0.004), and were more commonly chronic alcoholics (15 vs. 3.4%; *P* = 0.022). There was no significant between-group difference in the proportions of patients with cavitary lesions evident on initial chest X-ray or whose initial sputum specimens were AFB smear- and culture-positive, suggesting a high initial TB disease burden. Also, frequency and interval of sputum smear and culture test did not show significant difference between DIH and non-DIH groups.

**Table 1:** Demographic and clinical characteristics of the study population

Characteristic	Total (*N* = 168)	DIH (*n* = 20)	Non-DIH (*n* = 148)	*P*-value
Age, years	53.4 ± 19.2	62.9 ± 17.1	52.1 ± 19.2	0.018
Sex, male	111 (66.1)	11 (55)	100 (67.6)	0.267
Body mass index, kg/m^2^	21.2 ± 3.2	20.2 ± 3.6	21.4 ± 3.1	0.215
Charlson comorbidity index score	0.48 ± 0.67	0.80 ± 0.83	0.43 ± 0.63	0.042
Chronic liver disease	14 (8.3)	5 (25.0)	9 (6.1)	0.004
Chronic alcoholic	8 (4.8)	3 (15.0)	5 (3.4)	0.022
Diabetes mellitus	28 (16.7)	3 (15.0)	25 (16.9)	0.832
Cavitary lesion evident in chest X-ray	66 (39.3)	7 (35.0)	59 (39.9)	0.677
Frequency of sputum smear/culture	6.7 ± 3.1	6.6 ± 2.5	6.7 ± 3.1	0.968
Interval of sputum smear/culture, days	41.4 ± 18.3	41.4 ± 17.9	41.4 ± 18.4	0.975
Positive sputum AFB smear	80 (47.6)	10 (50.0)	70 (47.3)	0.821
Positive sputum AFB culture (liquid)	162 (96.4)	19 (95.0)	143 (96.6)	0.715
Positive sputum AFB culture (solid)	156 (92.9)	20 (100)	136 (91.9)	0.188
Positive sputum TB PCR (*n* = 65)	52 (80.0)	8 (80.0)	44 (80.0)	0.279

Data are presented as n (%) or means ± SDs. *DIH* drug-induced hepatotoxicity, *AFB* acid-fast bacilli, *PCR* polymerase chain reaction

### Risk factors for DIH

The univariate logistic regression analysis seeking factors affecting DIH development showed that age (OR 1.03; 95% CI 1.00–1.06; *P* = 0.022), a history of chronic liver disease (OR 5.15; 95% CI 1.53–17.4; *P* = 0.008), and the Charlson Comorbidity Index score (OR 2.07; 95% CI 1.10–3.88; *P* = 0.024) were significantly associated with DIH (Table 2). However, on subsequent multivariate analysis, only age and a history of chronic liver disease were independent risk factors for DIH; elderly patients and those with chronic liver disease were 1.03- and 4.51-fold more likely to develop DIH during TB treatment, respectively (95% CI 1.002–1.06, *P* = 0.036; 95% CI 1.31–15.55, *P* = 0.017).

**Table 2:** Risk factors for DIH

Characteristic	Univariate analysis		Multivariate analysis	
	OR (95% CI)	*P*-value	aOR (95% CI)	*P*-value
Age, years	1.03 (1.00–1.06)	0.022	1.03 (1.002–1.06)	0.036
Sex, male	0.59 (0.23–1.51)	0.269		
Chronic liver disease	5.15 (1.53–17.4)	0.008	4.51 (1.31–15.55)	0.017
Chronic alcoholic	5.05 (1.11–23.0)	0.036		
Diabetes mellitus	0.87 (0.24–3.19)	0.831		
Charlson comorbidity index score	2.07 (1.10–3.88)	0.024		

### Clinical outcomes by DIH status

DIH did not significantly influence the absolute treatment duration or the ratio of actual to planned treatment duration (Table 3). However, the ratio of treatment interruption time to total treatment duration was significantly higher in the DIH than the non-DIH group (0.04 ± 0.06 vs. 0.01 ± 0.04; *P* <  0.001). Patients with DIH were assessed in terms of hepatotoxicity severity using the DILIN criteria and reclassified into mild (13, 65%), moderate (3, 15%), and moderate-to-severe groups (4, 20%). The latter group experienced a significantly longer total treatment duration than the other groups (335.3 ± 114.0 vs. 228.6 ± 58.6 and 191.0 ± 12.5 days; *P* = 0.032) but did not differ significantly in terms of the ratio of actual to planned treatment duration or the ratio of the treatment interruption time to total treatment duration.

**Table 3:** Clinical outcomes of patients with TB by DIH status

Characteristics	Total (*N* = 168)	DIH (*n* = 20)	Non-DIH (*n* = 148)	*P*-value
Total treatment duration, days	243.4 ± 97.5	243.9 ± 81.5	243.3 ± 99.7	0.964
Actual treatment duration/planned treatment duration	1.14 ± 0.51	1.26 ± 0.48	1.13 ± 0.52	0.392
Treatment interruption period/total treatment duration	0.02 ± 0.06	0.04 ± 0.06	0.01 ± 0.04	< 0.001
Sputum culture conversion (solid) (*n* = 155)	155 (99.4)	20 (100.0)	135 (99.3)	0.587
Sputum culture conversion (liquid) (*n* = 162)	158 (98.1)	19 (100.0)	140 (97.9)	0.522
Time to sputum culture conversion (solid), days (*n* = 155)	47.0 ± 31.3	47.1 ± 26.1	47.0 ± 32.1	0.993
Time to sputum culture conversion (liquid), days (*n* = 162)	67.2 ± 96.8	64.0 ± 56.0	67.6 ± 101.2	0.859
2-month sputum culture conversion (solid) (*n* = 155)	129 (82.7)	16 (80.0)	113 (83.1)	0.787
2-month sputum culture conversion (liquid) (*n* = 162)	117 (73.1)	13 (68.4)	104 (73.8)	0.658
Treatment outcome				
Treatment completion	106 (63.1)	14 (70.0)	92 (62.2)	0.495
Cure	54 (32.1)	3 (15.0)	51 (34.5)	0.080
Treatment success	160 (95.2)	17 (85.0)	143 (96.6)	0.022
TB-related mortality	0 (0)	0 (0)	0 (0)	1.0
All-cause mortality	8 (4.8)	3 (15.0)	5 (3.4)	0.055
Recurrence within 1 year (*n* = 148)	7 (4.2)	2 (10)	5 (3.4)	0.196

Data are presented as n (%) or mean ± SD. *DIH* drug-induced hepatotoxicity, *TB* tuberculosis

**Fig. 2:**
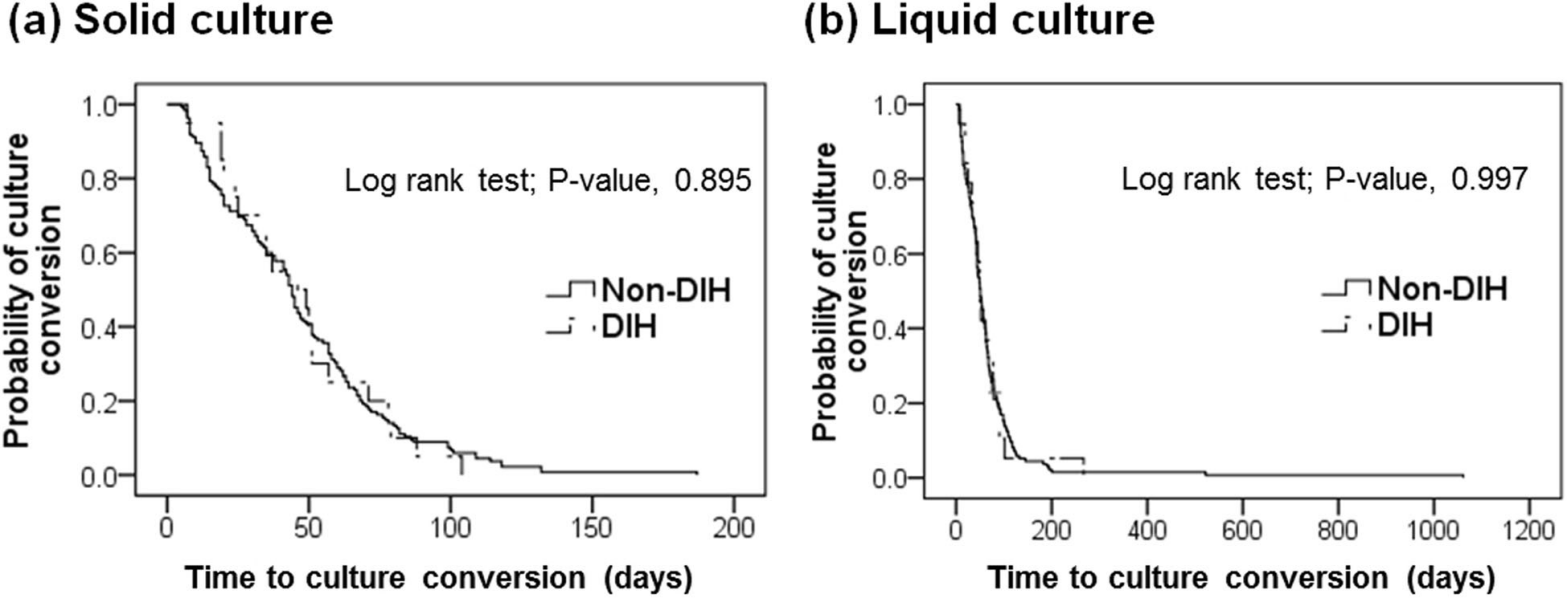
Kaplan–Meier curve of the time to sputum culture conversion by DIH development. Non-DIH group is presented as solid line, and DIH group as chain line

In terms of follow-up sputum AFB cultures, the rates of negative culture conversion within 2 months and during the entire treatment period, as well as the time to negative culture conversion, were not significantly affected by the development of DIH. These results were consistent with those of Kaplan–Meier analysis used to determine whether DIH occurrence influenced the time to negative culture conversion of sputum specimens (solid culture, *P* = 0.895; liquid culture, *P* = 0.997) (Fig. 2).

In terms of treatment outcomes, the rates of treatment completion, cure, and all-cause mortality did not differ by DIH status, but the treatment success rate was significantly lower in the DIH group (85% vs. 96.6%; *P* = 0.022). We recorded no instance of TB-related mortality, treatment failure, or acquired drug resistance. Of patients who had been followed-up for more than 1 year after treatment, recurrence within 1 year was somewhat more common in the DIH group, but the difference was not statistically significant (10 vs. 3.4%; *P* = 0.196). On multivariable analysis of factors associated with treatment success and sputum AFB culture conversion within 2 months, the only significant factor was smear-positivity of the initial sputum specimen, which serves as the index of the initial disease burden (Table 4), significantly compromising both treatment success and sputum AFB culture conversion within 2 months (aHR 0.60, 95% CI 0.43–0.83, *P* = 0.002; aHR 0.52, 95% CI 0.35–0.78, *P* = 0.001). DIH was not significantly associated with these parameters after adjustment for other clinical factors.

**Table 4:** Factors contributing to treatment success and sputum liquid culture conversion within 2 months (multivariable analysis)

Characteristic	Treatment success		Sputum liquid culture conversion within 2 months	
	aHR (95% CI)	*P*-value	aHR (95% CI)	*P*-value
Age, years	0.99 (0.98–1.00)	0.166	1.00 (0.99–1.01)	0.597
Sex, male	0.99 (0.69–1.42)	0.951	1.30 (0.86–1.97)	0.210
Chronic liver disease	0.80 (0.42–1.50)	0.478	0.77 (0.38–1.58)	0.478
Charlson comorbidity index score	0.89 (0.65–1.22)	0.473	0.96 (0.67–1.38)	0.827
Cavitation evident in initial chest X-ray	0.71 (0.50–1.03)	0.069	0.81 (0.54–1.22)	0.310
Smear-positivity of initial sputum specimen	0.60 (0.43–0.83)	0.002	0.52 (0.35–0.78)	0.001
Drug-induced hepatotoxicity	0.97 (0.57–1.65)	0.915	0.89 (0.48–1.62)	0.692

When additional analysis was performed only for DIH patients, reclassification using the DILIN criteria was not associated with any significant difference in the 2-month sputum culture conversion or treatment success rate, or DIH severity.

### Management of DIH

Table 5 lists the DIH management methods employed. Of the 20 DIH patients, the DIH onset time was a mean of 61.9 days, ranging from 6 to 204 days after initiation of TB treatment. Nine patients (45%) were diagnosed with DIH within 30 days of treatment commencement. The DIH group exhibited a higher proportion of patients taking hepatotonics than the non-DIH group (85 vs. 16.2%; *P* <  0.001), In addition, DIH patients took a greater variety of hepatotonics for a significantly longer period during TB treatment compared with the non-DIH group. In the DIH group, hepatotonics were taken for an average of 37% of the total treatment duration.

**Table 5:** Management of DIH

*N* = 168	DIH (*n* = 20)	Non-DIH (*n* = 148)	*P*-value
Onset time of DIH from treatment commencement, days	61.9 ± 60.3		
Hepatotonics			
Administration	17 (85.0)	24 (16.2)	< 0.001
Drug number	1.40 ± 0.75	0.19 ± 0.46	0.001
Medication use rate	0.37 ± 0.29	0.06 ± 0.17	< 0.001
Re-introduction methods			
Recommence full-dose drugs after 1 week	8 (40.0)		
Regimen change	7 (35.0)		
Start with low dose drugs followed by stepwise increases and addition of more drugs	3 (15.0)		
Addition of full-dose drugs at intervals of 1 week	2 (10.0)		

Data are presented as n (%) or means ± SDs. *DIH* drug-induced hepatotoxicity

When recommencing TB medications after a pause caused by DIH, several re-introduction methods were used. The most common approach (eight patients, 40%) was to restart full-dose drugs after 1 week. A regimen change was the second most preferred method (seven patients, 35%). The clinical outcomes did not differ by the re-introduction method employed.

## Discussion

DIH developed during treatment of 11.9% of patients with culture-proven, drug-susceptible pulmonary TB. This incidence is slightly higher than that reported in previous South Korean studies (8.7–10.5%) [5, 6, 19]; the incidence of chronic liver disease (8.2%) was also similar to those of previous studies [5, 6]. Our population had a lower body mass index (21.2 ± 3.2 kg/m^2^) than did patients of earlier studies, possibly increasing the risks of DIH [10, 20, 21]. The DIH incidence in patients with chronic liver disease was 35.7%; older age and chronic liver disease were independent risk factors, in line with the findings of previous studies [19, 22, 23].

DIH did not influence total treatment duration or sputum culture conversion, but was associated with significant changes in treatment interruption time and the success of TB eradication. In one study of patients receiving the standard TB regimen, an intermittently treated group relapsed significantly more often after treatment than did a daily treatment group [24]. This was true when intermittent treatment was confined to either the continuation or intensive phase. This suggests that a lower total drug dose during the planned treatment period increases the risk of recurrence. In this context, treatment interruption caused by DIH may create a risk of future TB relapse. We found that DIH developed predominantly within 2 months of treatment initiation; 45% of DIH cases occurred within the first 30 days. It is possible that TB may be undertreated in the intensive phase when treatment is interrupted because of DIH. When drugs are recommenced after DIH, treatment with subtherapeutic doses may create risks of future relapse and acquired resistance. Regimen changes were employed in 35% of re-introductions, at least partially varying the standard regimen, replacing effective drugs with less-effective drugs. This may explain the higher rate of recurrence in the DIH group within 1 year compared with the non-DIH group, although this difference was not statistically significant (10 vs. 3.4%).

We found that the success rate of TB treatment was lower in the DIH than the non-DIH group (85 vs. 96.6%), consistent with a prior Chinese study [25]. The difference seems to be attributable to a higher all-cause mortality rate in the former group (15 vs. 3.4%). In the DIH group, the higher numbers of patients who were elderly and/or exhibited comorbidities may have negatively impacted treatment outcomes and mortality. It is possible that our statistical power was inadequate to capture other effects on these outcome variables because of the relatively small number of subjects.

Although many studies have sought risk factors for DIH, no generally accepted effective treatment is available. It is unclear whether hepatotonics improve DIH [26–28]. In this study, 85.0% of DIH patients took hepatotonics, but we could not confirm that these affected the TB clinical outcomes However, DIH increases socioeconomic health costs because of the need for additional medicines, potential adverse effects, and the requirement for admission to treat DIH.

Currently, there is no standard method for re-introduction of anti-TB medications after DIH develops. A previous study found no significant difference in the DIH recurrence rate when several re-introduction methods were employed, including recommencement of full-dose drugs, addition of full-dose drugs at intervals of 1 week, and initiation of low-dose drugs followed by stepwise increases and addition of more drugs [29]. We found that various re-introduction methods were used in the absence of bias; however, our evaluation of the efficacy and safety of the re-introduction methods was limited by the number of study subjects.

Our work had several limitations. This was a retrospective study with a limited level of evidence. All data were collected at a single center. As mentioned, to precisely evaluate the impact of DIH on the clinical course, we excluded patients for whom the treatment response was difficult to assess; i.e., patients lost to follow-up, who transferred, and who died early. Also, all study patients were initially treated with the standard regimen. Therefore, our findings should be generalized only with caution.

## Conclusion

DIH development was common during TB treatment, and was associated with older age and a history of chronic liver disease, but did not significantly affect the sputum culture conversion, treatment outcome, or total TB treatment duration. However, treatment interruption caused by DIH may increase the risk of future relapse and acquired drug resistance. DIH management is not well established, and further research is required.

## Data Availability

The datasets analyzed during the current study are not publicly available, but are available from the corresponding author on reasonable request.

## Abbreviations

AFB: Acid-fast bacilli
ALT: Alanine aminotransferase
aOR: Adjusted odds ratio
AST: Aspartate aminotransferase
CI: Confidence interval
DIH: Drug-induced hepatotoxicity
DILIN: Drug-Induced Liver Injury Network
OR: Odds ratio
PCR: Polymerase chain reaction
TB: Tuberculosis
ULN: Upper limit of normal
